# Natural Product-Inspired Dopamine Receptor Ligands

**DOI:** 10.1021/acs.jmedchem.4c00537

**Published:** 2024-07-22

**Authors:** Michael Dorogan, Hari K. Namballa, Wayne W. Harding

**Affiliations:** † Department of Chemistry, Hunter College, City University of New York, 695 Park Avenue, New York, New York 10065, United States; ‡ Program in Biochemistry, CUNY Graduate Center, 365 Fifth Avenue, New York, New York 10016, United States; § Program in Chemistry, CUNY Graduate Center, 365 Fifth Avenue, New York, New York 10016, United States

## Abstract

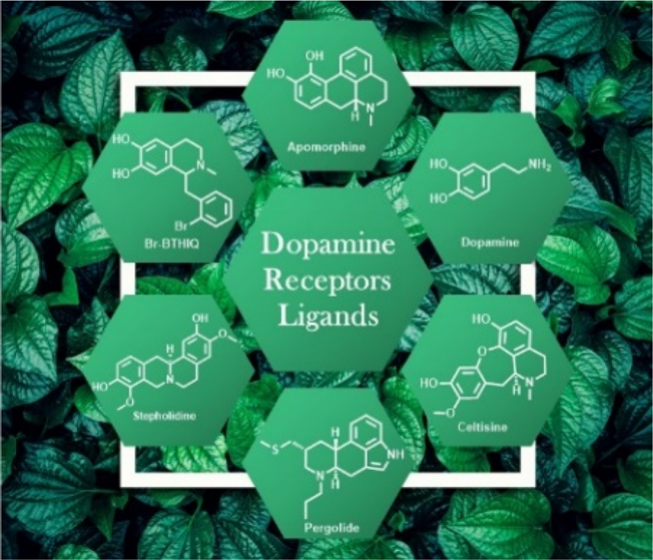

Due to their evolutionary bias as ligands for biologically relevant drug targets, natural products offer a unique opportunity as lead compounds in drug discovery. Given the involvement of dopamine receptors in various physiological and behavioral functions, they are linked to numerous diseases and disorders such as Parkinson’s disease, schizophrenia, and substance use disorders. Consequently, ligands targeting dopamine receptors hold considerable therapeutic and investigative promise. As this perspective will highlight, dopamine receptor targeting natural products play a pivotal role as scaffolds with unique and beneficial pharmacological properties, allowing for natural product-inspired drug design and lead optimization. As such, dopamine receptor targeting natural products still have untapped potential to aid in the treatment of disorders and diseases related to central nervous system (CNS) and peripheral nervous system (PNS) dysfunction.

## 1 Introduction

Isolated from natural sources as byproducts of primary or secondary metabolism, natural products are important conduits in drug discovery as lead compounds.^1^ The significance of natural products in pharmaceuticals cannot be overstated as over 30% of all FDA-approved new molecular entities (NMEs) are derived directly or inspired from microbial and plant species.^2^ Optimized by natural selection pressures, natural products are refined to have optimal interactions with biological macromolecules, membrane permeability, biomolecular compatibility, and stability, thus sharing key properties of drug likeness.^3,4^ A recent analysis revealed that over 80% of CNS agents are derived directly or inspired from natural products, and the scaffolds of just 20 natural products yielded more than 400 clinically approved CNS drugs.^5^

Characteristically defined by their seven transmembrane domains, G protein-coupled receptors (GPCRs) are a superfamily of evolutionarily related integral membrane proteins. GPCRs are canonically coupled to heterotrimeric G proteins, consisting of three associated protein subunits (α, β, and γ). Activation of GPCRs results in the dissociation of the heterotrimeric G proteins into α-subunit and βγ-complex, further transducing the signal to stimulate the effector systems (which are dependent on the specific type of G protein). It should be noted that other signaling mechanisms (e.g., involving ion channels, receptor tyrosine kinases, or β-arrestins) are now known to exist. GPCRs are a critical drug target due to their implication in a wide range of diseases (including cancers, inflammatory diseases, mental disorders, metabolic disorders, cardiovascular diseases, and sensory disorders). In fact, over 30% of FDA-approved drugs target GPCRs.^6^

**Figure 1:**
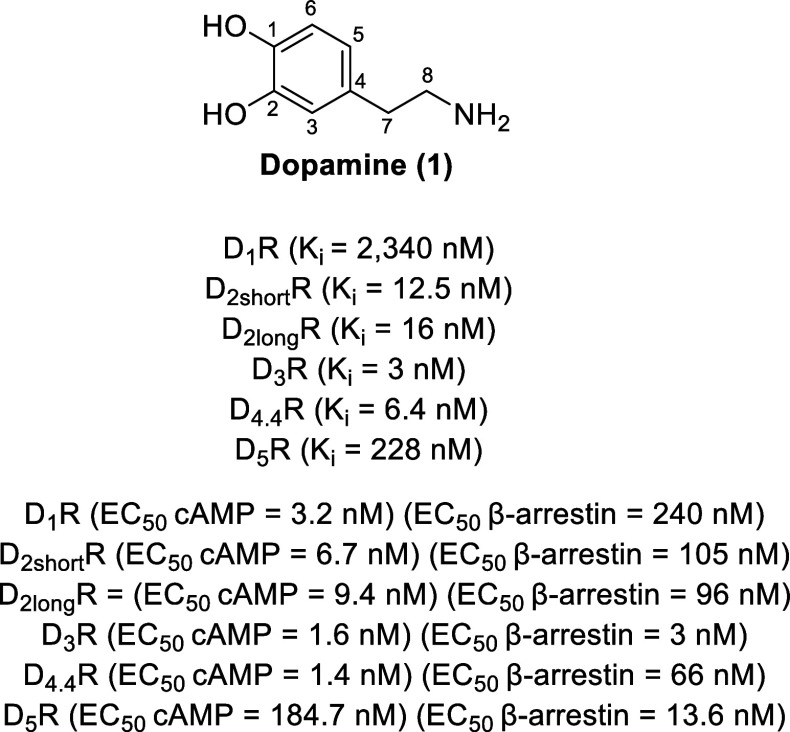
Structure and dopamine receptor affinities and activities of dopamine.

Dopamine receptors are GPCRs that recognize the endogenous, catecholamine dopamine (**1**, Figure 1). The five subtypes of dopamine receptors are categorized into two families based on whether they stimulate cAMP production (the D_1-like_ family: D_1_R and D_5_R subtypes) via activating Gα_s_ or Gα_olf_ proteins or attenuate cAMP production (the D_2-like_ family: D_2_R, D_3_R, and D_4_R subtypes) via activating Gα_i/o_ proteins (Table 1).^7−9^ It should be noted that two splice variants of the D_2_R occur (D_2short_R or D_2S_R and D_2long_R or D_2L_R), where D_2L_R has a 29-mer peptide insertion in the third intracellular loop. These splice variants are predominantly localized to pre- and postsynaptic membranes, respectively.^10^

**Table 1:** Characteristics of Dopamine Receptors

	D_1-like_ family		D_2-like_ family		
	D_1_R	D_5_R	D_2_R	D_3_R	D_4_R
expression	high levels of expression: mesocortical mesolimbic, and nigrostriatal areas	adrenal glands, blood vessels, cortex, dental gyrus, GI tract, heart, hippocampus, hypothalamus, kidneys, substantia nigra, sympathetic ganglia	high levels of expression: caudate, nucleus accumbens, olfactory bulb putamen, striatum, substantia nigra, tubercle, and ventral tegmental area	hippocampus, hypothalamus, nucleus accumbens, olfactory bulb, striatum, and substantia nigra	adrenal glands, amygdala, blood vessels, frontal cortex, GI tract, heart, hippocampus, hypothalamus, kidneys, mesencephalon, nucleus accumbens, substantia nigra, sympathetic ganglia, and thalamus
	low levels of expression: cerebellum, hippocampus, hypothalamic areas, kidneys, and thalamus		low levels of expression: adrenal glands, blood vessels, cortex, GI tract, heart, hypothalamus, septum, kidneys, and sympathetic ganglia		
functions	attention, control of rennin in kidney, impulse control, locomotor activity, regulation of feeding, regulation of memory and learning, reproductive behaviors, reward and reinforcement, sleep	affective functions, endocrine functions, pain process	GI motility, regulation of aldosterone and prolactin secretion, regulation of blood pressure, renal functions, reward and reinforcement, vasodilatations, working memory	cognition, emotions, endocrine functions, regulation of locomotor functions	GI motility, modulations of cognitive functions, regulation of renal functions, vasodilatations, blood pressure,
(potential) therapeutic applications	addiction/substance abuse disorder, antihypertensive, obesity, Parkinson’s disease, schizophrenia, Tourette’s syndrome		antipsychotics, depression, Parkinson’s disease, schizophrenia	addiction/substance abuse disorder, Parkinson’s disease, schizophrenia	addiction/substance abuse disorder, ADHD, neuropsychiatric disorders, sexual dysfunction
associated adverse drug reactions		cardiovascular side effects	hyperprolactinemia, metabolic dysfunction	extrapyramidal motor symptoms, hyperprolactinemia, metabolic dysfunction	metabolic dysfunction

In addition to canonical signaling via G proteins, dopamine receptors are also known to signal via β-arrestin-based pathways. This (relatively recent) recognition of alternative signal transduction pathways has opened up the possibility to discover ligands that are selective for either G protein or β-arrestin pathways (so-called “functionally selective” or “biased ligands”), leading to different downstream effects.^9,11−15^ Functional selectivity broadens the conventional definitions of pharmacology to expand the possibility of ligands to act with a mixture of classic characteristics (agonist, antagonist, and/or inverse agonist) depending on the effector pathway.^16,17^ In other words, functional selectivity describes the ability of a particular ligand to differentially activate distinct subsequent signaling cascades or the selective activation of a specific G protein subtype.^18−20^ This allows for the potential to mediate divergent processes or the ability to bypass observed adverse effects through the activation of distinct signaling pathways.^21−25^

As the physiological functions of dopamine are quite extensive (including functions such as reward, sleep regulation, voluntary movement, penile erection, sympathetic regulation, cognitive function, olfaction, and hormonal regulation), dopamine receptors are attractive drug targets.^26^

**Figure 2:**
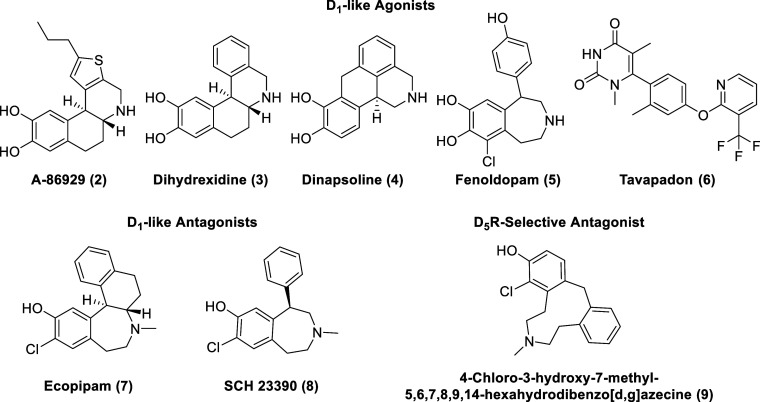
Representative selective agonists and antagonists of D_1-like_ receptors.

At this time, no selective D_1_R agonist has been commercialized as a CNS-marketed drug. Traditional D_1_R agonists, such as A-86929 (**2**, Figure 2), dihydrexidine (**3**), and dinapsoline (**4**), are characterized by a catechol moiety as typified by dopamine. These ligands have poor CNS penetration and oral bioavailability due to the polarity of the catechol moiety.^27,28^ Fenoldopam (**5**), a synthetic benzazepine derivative, is an FDA-approved D_1-like_ agonist, but due to its short duration of action and poor pharmacokinetics, fenoldopam use is limited to emergency medicine as an IV injection in the treatment of hypertensive crisis.^29−31^ Fenoldopam’s mechanism of action relies on the activation of peripheral D_1-like_ receptors (no blood–brain barrier (BBB) permeability), which results in the reduction of systemic arterial blood pressure through renal vasodilation.^32^

The high degree of structural homology between the ligand binding site of D_1_R and D_5_R further exacerbates the difficulties in obtaining selective D_1_R ligands. This may be of therapeutic relevance since D_5_R is associated with cardiovascular side effects.^33^ Nevertheless, D_1_R is an important target for the treatment of cognitive deficits, particularly those associated with schizophrenia (which are not currently addressed by antipsychotic medications) and Parkinson’s disease. This is because full or partial agonists of D_1_-like receptors have been shown to reverse working memory in aged monkeys or from deficits induced by ketamine administration.^34−36^

Relatively recently, researchers at Pfizer identified a new class of potent noncatechol D_1-like_ orthosteric agonists through a high-throughput screening (HTS) campaign of approximately 3 million compounds and subsequent structure–activity relationship (SAR) studies.^37,38^ The clinical efficacity of these agonists are still being evaluated with the optimized drug candidate, Tavapadon (**6**, *K*_i_ D_1_R = 8.54 nM), currently under phase 3 clinical trials for Parkinson’s disease in the United States (under development by Cerevel Therapeutics) [NCT04201093, NCT04223193].^39,40^

D_1_ antagonism may also hold therapeutic promise as well, as exemplified by Ecopipam (SCH 39166, **7**). Ecopipam, a more conformationally restricted benzazepine analog of SCH 23390 (**8**, the most used D_1_R reference antagonist), is a selective D_l_-like dopamine receptor antagonist (in vitro and in vivo) (*K*_i_ at D_1_R of 1.2 nM, *K*_i_ at D_5_R of 2 nM) with improved selectivity over 5-HT_2_ and duration of action.^41^ Ecopipam displays a poor pharmacokinetic profile with extensive *N*-dealkylation of the *N*-methyl group and *O*-glucuronidation of the phenol as well as low oral bioavailability (0.6%).^42,43^ Ecopipam was initially studied as a treatment for schizophrenia, but it displayed no efficacy (with reported side effects of anxiety, restlessness, sedation, and vomiting).^44,45^ Clinical studies for cocaine addiction revealed that while Ecopipam is an effective antagonist of the acute euphoric effects of cocaine, repeated administration failed to attenuate the subjective effects of cocaine.^46,47^ Clinical studies also revealed that Ecopipam is an effective obesity treatment; however, undesirable side effects (depression and reversible anxiety) halted its development as an antiobesity drug.^48,49^ Ecopipam is currently under phase 3 clinical trials for Tourette’s syndrome in the United States [NCT06021522, NCT05615220].^50,51^

**Figure 3:**
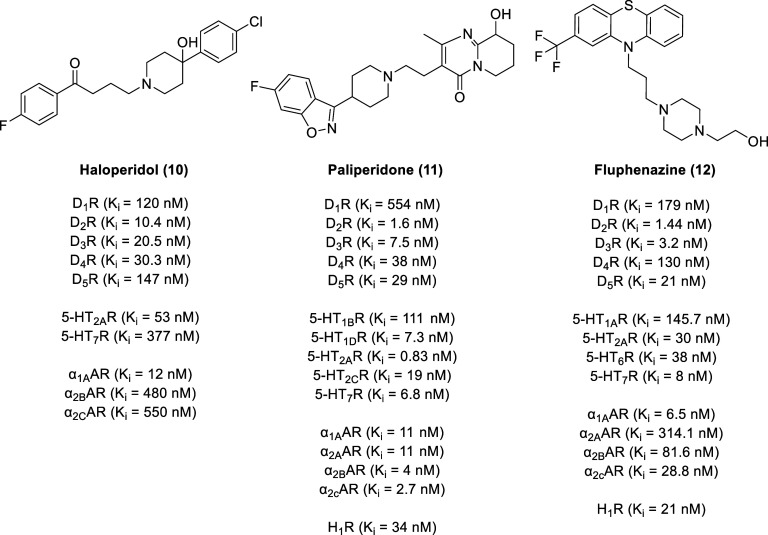
Representative nonselective D_2-like_ receptor agonists.

As one of the most established therapeutic targets for endocrine and neuropsychiatric disorders, the D_2-like_ family of dopamine receptors are promising therapeutic targets. Despite their importance, most investigational and clinically approved drugs are not subtype selective but rather are generally subfamily selective for all three D_2_-like receptors (D_2_R, D_3_R, and D_4_R) due to the high degree of similarity between the transmembrane (TM) regions.^52^ For example, antipsychotic drugs such as haloperidol (Haldol, **10**, Figure 3), paliperidone (Invega, **11**), and fluphenazine (Prolixin, **12**) not only function as D_2-like_ receptor antagonists but also engage serotonin and adrenergic receptors.^53−57^

Due to the prevalence of dopamine D_2-like_ signaling in the periphery (mainly in the pancreatic α- and β-cells), D_2-like_ ligands often display metabolic dysfunction (e.g., systemic insulin resistance and dysglycemia).^58^ Moreover, side effects can result from antagonism of D_2_R signaling in the nigrostriatal system, causing extrapyramidal motor symptoms, and in the tuberoinfundibular pathway, resulting in elevated prolactin levels.^9,59^

**Figure 4:**
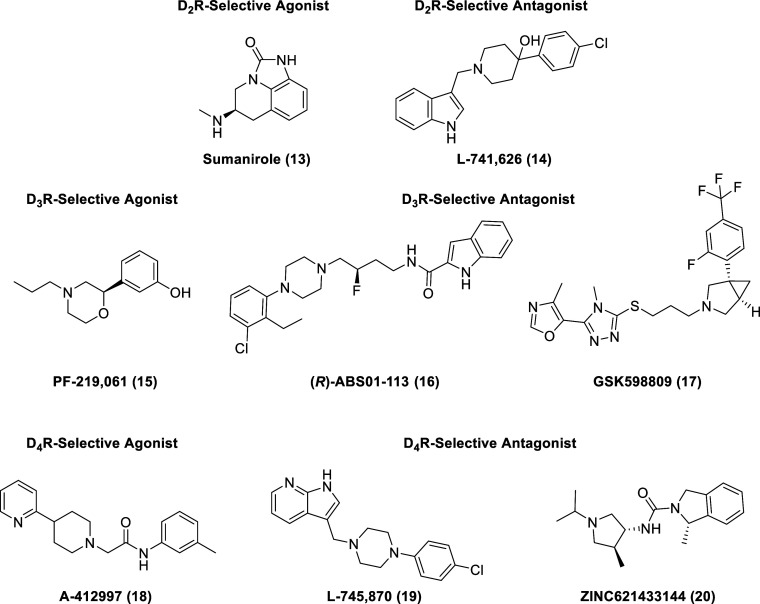
Representative selective agonists and antagonists of D_2-like_ receptors.

Nevertheless, subtype-selective ligands for each D_2-like_ subtype (representative examples shown, **13**–**20**, in Figure 4) are proven to have significant therapeutic applications. All FDA-approved antipsychotics are D_2_R partial agonists or antagonists. In addition, D_2_R ligands are used in the treatment of restless legs syndrome, postoperative nausea and vomiting, hyperprolactinemia, and Tourette’s syndrome. D_3_R ligands are implicated in neurological disorders and are being investigated as potential treatments for substance use disorders.^60,61^ D_4_R agonists are proposed therapeutic agents for attention deficit hyperactivity disorder (ADHD) and sexual dysfunction, while D_4_R antagonists are candidates for the treatment of neuropsychiatric disorders.^62,63^

Despite their clinical use, conventional dopamine receptor targeting drugs have debilitating side effects mainly due to suboptimal pharmacokinetic and pharmacodynamic properties. The identification of new chemotypes for dopamine receptor ligand discovery and the refinement of existing chemotypes are avenues that may lead to novel and pharmacologically advanced therapeutics. Natural products such as l-DOPA, benzyltetrahydroisoquinolines, aporphine alkaloids, tetrahydroprotoberberines, and ergolines have inspired and will continue to stimulate dopamine receptor ligand discovery efforts in that regard. Herein, we provide an informed perspective on the status of the aforementioned structural classes as a source of ligands for dopamine receptors.

## 2 Dopamine and l-DOPA

**Figure 5:**
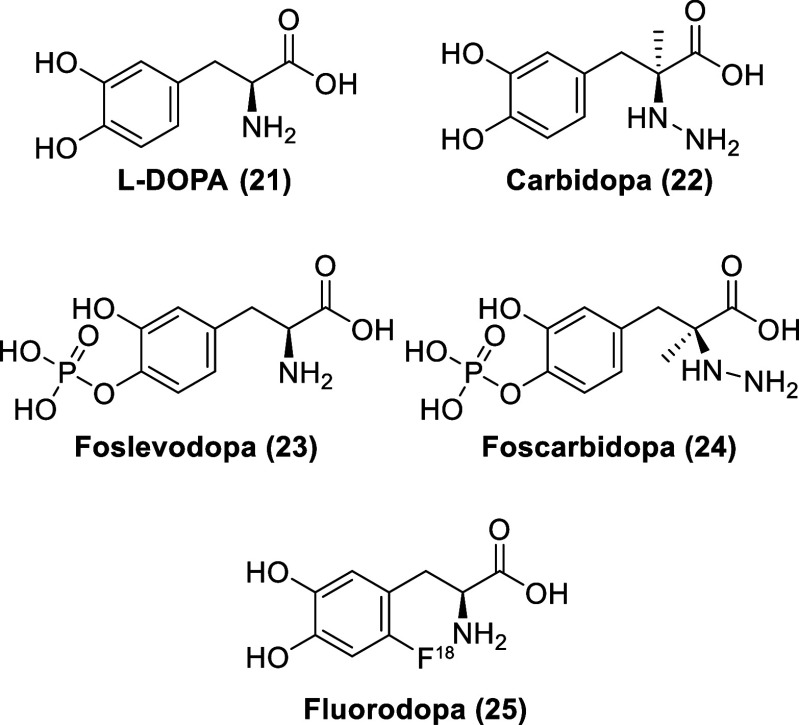
Chemical structures of l-DOPA, carbidopa, and related compounds.

The endogenous neurotransmitter dopamine (**1**, Figure 1) functions to activate dopamine receptors with the affinity trend of D_3_R (*K*_i_ = 3 nM) > D_4.4_R (*K*_i_ = 6.4 nM) > D_2S_R (*K*_i_ = 12.5 nM) > D_2L_R (*K*_i_ = 16 nM) ≫ D_5_R (*K*_i_ = 228 nM) ≫ D_1_R (*K*_i_ = 2340 nM) (D_4.4_R is a human polymorphic variant).^64−67^ While dopamine cannot be used as a CNS drug, as it cannot cross the protective BBB, it can still be used as a peripheral vasostimulant for the treatment of low heart rate, cardiac arrest, and low blood pressure.^68,69^ Originally believed to be a biologically inactive amino acid, l-3,4-dihydroxyphenylalanine (l-DOPA, **21**, Figure 5) was isolated from seedlings of *Vicia faba* beans in 1913.^70,71^l-DOPA is transported across the BBB by the large neutral amino acid transporter (LAT1) and is subsequently decarboxylated to dopamine by the endogenous enzyme aromatic l-amino acid decarboxylase. l-DOPA bypasses the rate-limiting step in dopamine synthesis (involving the enzyme tyrosine hydroxylase) and is more quickly converted to dopamine than the biosynthetic starting material for dopamine production, the amino acids l-tyrosine or l-phenylalanine. As such, l-DOPA is able to increase dopamine concentrations and reduce the motoric symptoms of Parkinson’s disease.^72^ Parkinson’s disease is a long-term CNS degenerative disorder, characterized by the idiopathic loss of dopamine neurons in the caudate-putamen.^73^ The loss of these neurons causes the manifestation of Parkinson’s disease motor symptoms, characterized by rigidity, slowed movements, difficulty with walking, and tremors.

Most patients treated with l-DOPA will eventually develop l-DOPA-induced dyskinesia and motor fluctuations due to dysfunction in dopaminergic pathways.^72^ Particularly, dyskinesia is believed to be caused by pathological alterations in the nigrostriatal pathway.^74^ It should be noted that l-DOPA will increase dopamine concentration not just in the CNS but also in the peripheral nervous system (yielding undesirable side effects). As such, it is a standard clinical practice to coadminister a peripheral DOPA decarboxylase inhibitor (DDCI) (e.g., carbidopa, **22**) with l-DOPA to prevent the conversion of l-DOPA to dopamine in the periphery.^75^ But, even with the coadministration of carbidopa, levodopa still has a relatively short half-life in plasma.^76^ Moreover, gastric motility is typically impaired as Parkinson’s disease progresses, rendering the absorption of oral tablets of levodopa to be unpredictable due to erratic gastric emptying (levodopa is only absorbed in the superior part of the duodenum, the first part of the small intestine). Duopa (AbbVie), a gel formulation of levodopa and carbidopa, allows for the prevention of the peaks and troughs associated with traditional levodopa and carbidopa tablet therapy through continuous dosing of levodopa and carbidopa via a jejunal tube (requiring surgery at the beginning of the therapy).^77^ As an alternative, phosphonate prodrugs of levodopa (foslevodopa, **23**) and carbidopa (foscarbidopa, **24**) enable a more effective subcutaneous delivery with significant improvement of aqueous solubility at physiological pH (7.4).^78,79^

6-[^18^F]Fluoro-l-DOPA (FDOPA, **25**) is a radiolabeled analog of l-DOPA, used in positron emission tomography (PET) imaging of dopaminergic nerve terminals in the striatum in patients with suspected Parkinsonian syndromes.^80^

## 3 Benzylisoquinoline Alkaloids (BIAs)

**Figure 6:**
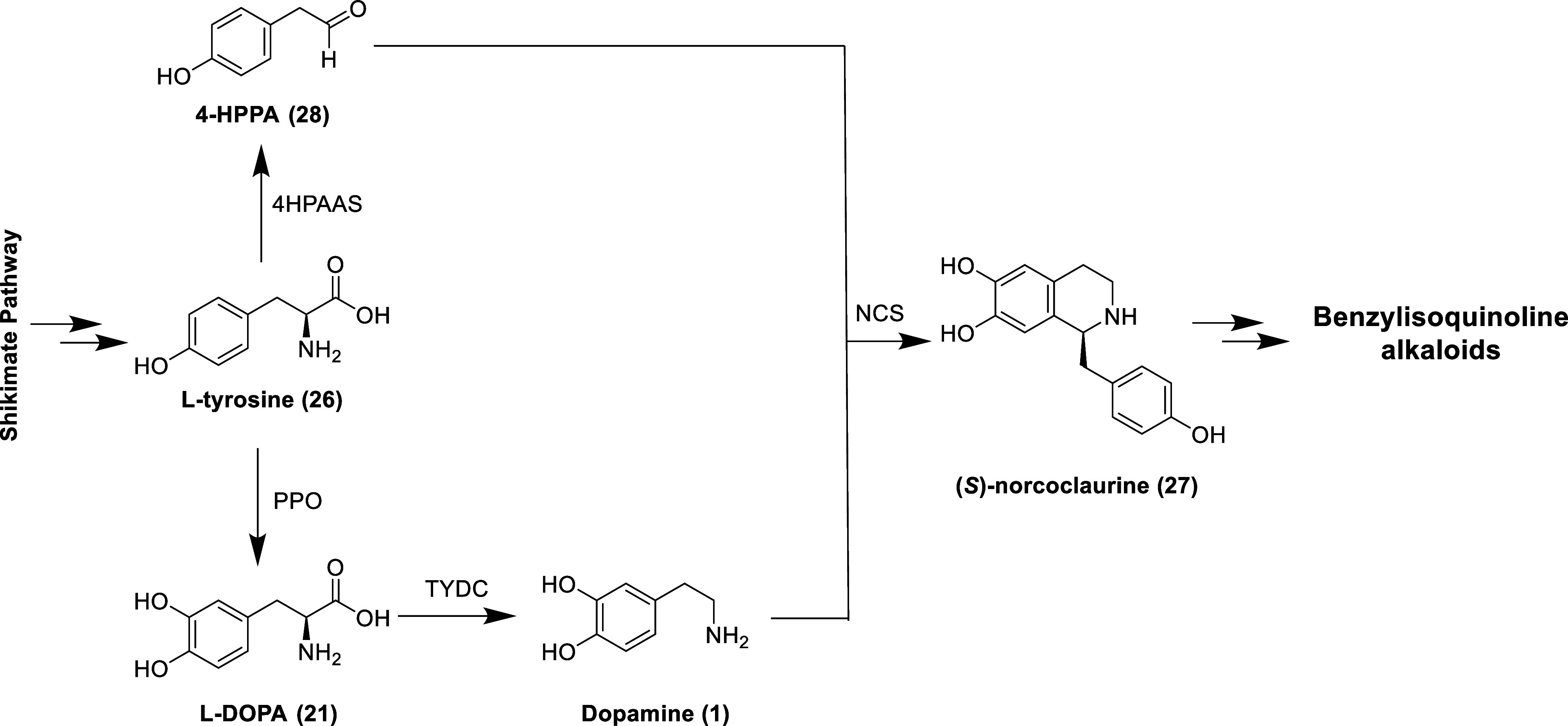
Biosynthesis of (*S*)-norcoclaurine, the common precursor to the benzylisoquinoline alkaloids.

**Figure 7:**
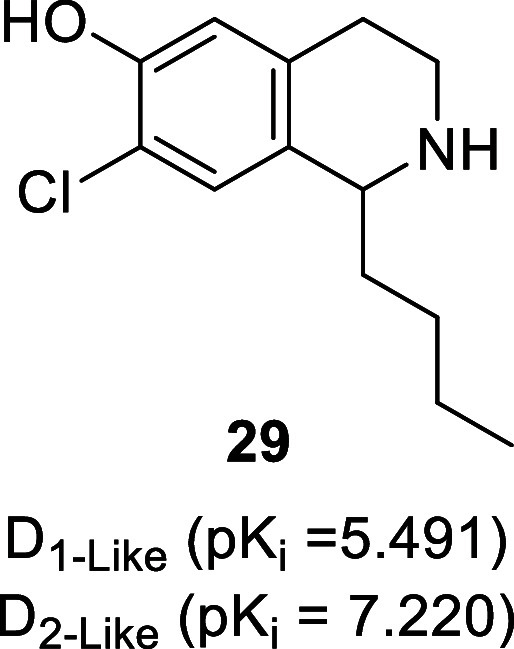
Structure of 1-butyl-7-chloro-6-hydroxy-tetrahydroisoquinoline.

Derived biosynthetically from the amino acid l-tyrosine (Tyr, **26**), the benzylisoquinoline alkaloids (BIAs) are a large structural group of plant secondary metabolites found primarily in the order Magnoliales, Laurales, Ranunculales, Berberidales, and Papaverales.^81^ (*S*)-Norcoclaurine (**27**, Figure 6) is the common precursor to the benzylisoquinoline derivatives (e.g., aporphine, benzophenanthridine, protoberberine, and pavine alkaloids) and is generated via the enantioselective condensation of dopamine and 4-hydroxyphenylacetaldehyde (4-HPPA, **28**) by the enzyme norcoclaurine synthase (NCS).^82^ 4-HPPA is produced directly from Tyr via decarboxylation–oxidative deamination by 4-hydroxyphenylacetaldehyde synthase (4HPAAS), whereas dopamine is formed in a two-step process (with l-DOPA as an intermediate) starting with the hydroxylation of Tyr by polyphenol oxidase (PPO) and subsequential decarboxylation by l-tyrosine decarboxylase (TYDC).^83^ As the BIAs are biogenetically derived from dopamine, it should not be surprising that several natural and synthetic benzylisoquinoline derivatives have dopaminergic activities as highlighted in the following subsections.^84^ The presence of the 1-benzyl moiety of BIAs is not essential for dopaminergic activity as seen with 1-butyl-7-chloro-6-hydroxy-tetrahydroisoquinoline (**29**, Figure 7, D_2-like_ receptors *K*_i_ = 66 nM), which displays antidepressant-like activity in mice.^85^

### Benzyltetrahydroisoquinolines (BTHIQs) and Bis-Benzyltetrahydroisoquinolines (BBTHIQs)

**Figure 8:**
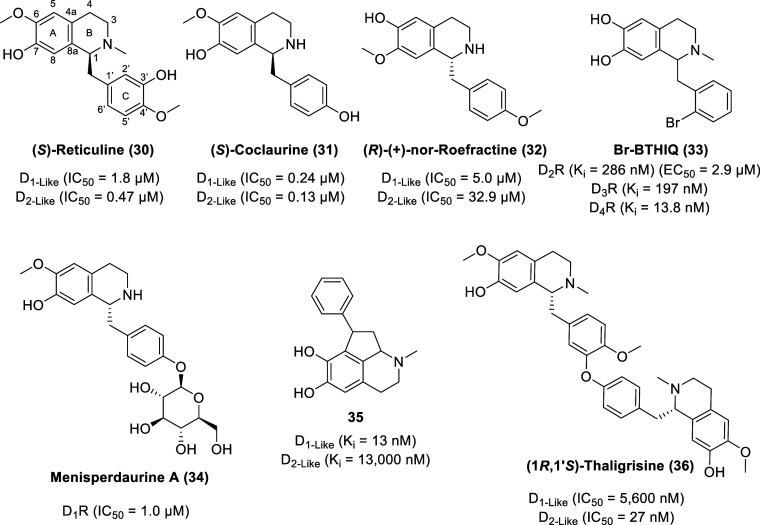
Chemical structures of selected BTHIQs and BBTHIQs.

Originally isolated from the roots of Bullock’s heart (*Annona reticulata*), (*S*)-reticuline (**30**, Figure 8) is a dopamine receptor ligand with low micromolar or submicromolar affinities to both D_1_ and D_2_ rat striatal receptors with IC_50_ values of 1.8 (D_1_-like receptors) and 0.47 μM (D_2_-like receptors).^86,87^ Ethanolic extracts of stems and roots of the evergreen shrub *Cocculus laurifolius* revealed (*S*)-coclaurine (**31**), a benzyltetrahydroisoquinoline with IC_50_ values of 0.24 (D_1_-like receptors) and 0.13 μM (D_2_-like receptors), based on the displacement of either tritiated raclopride (a D_2_ dopamine receptor-selective ligand) or SCH23390 (a D_1_ dopamine receptor-selective ligand) from their specific binding sites in rat striatum.^86,88^ (*R*)-(+)-Nor-roefractine (**32**) is a coclaurine analogue with a 4′-methoxy group (enhancing lipophilicity) and C-6 and C-7 substituents exchanged and displays 6-fold selectivity for D_2_ receptors although with lower potency compared to other benzyltetrahydroisoquinolines (BTHIQs). The IC_50_ values for the displacement of tritiated raclopride (D_2_-like receptors) or SCH23390 (D_1_-like receptors) by (*R*)-(+)-nor-roefractine are 5.0 and 32.9 μM, respectively.^89^

1-(2′-Bromobenzyl)-6,7-dihydroxy-*N*-methyl-tetrahydroisoquinoline (Br-BTHIQ, **33**) displays a partial agonist effect through cAMP signaling at D_2_R with an EC_50_ value of 2.9 μM (*E*_max_ = 31.9% at 10 μM, *E*_max_ = 48.4% at 100 μM), quantified using the homogeneous time-resolved fluorescence (HTRF)-based cAMP kit in Chinese hamster ovary (CHO)-K1 cells stably expressing the cloned human D_2S_R.^90^ Br-BTHIQ displays nanomolar affinity to dopamine receptors with the order of D_4_R (*K*_i_ = 13.8 nM) ≫ D_3_R (*K*_i_ = 197 nM) > D_2_R (*K*_i_ = 286 nM).

Menisperdaurine A (**34**) is a glycosidic benzylisoquinoline alkaloid isolated from the rhizomes of *Menispermum dauricum*, known as Bian-Fu-Ge-Gen or Bei-Dou-Gen in traditional Chinese medicine with analgesic and antipyretic effects.^91^ Menisperdaurine A displays D_1_R antagonistic activity with an IC_50_ value of 1.0 μM [via fluorometric imaging plate reader (FLIPR) assay, monitoring cellular Ca^2+^ responses].

Synthetic BTHIQ derivatives bearing a cyclopentane motif and a phenyl substituent display high selectivity and affinity toward D_2-like_ receptors as exemplified by **35**, affinity values (*K*_i_) of 13 nM at D_1-like_ receptors and 13 000 nM at D_2-like_ receptors (*K*_i_ D_1_/D_2_ = 1000).^92^

Bis-benzyltetrahydroisoquinolines (BBTHIQs) are biosynthetically generated by dimerization of trioxygenated benzyltetrahydroisoquinolines via intermolecular oxidative coupling of (*S*)- and/or (*R*)-coclaurine and/or its *N*-methyl derivatives.^84^ BBTHIQs with one diaryl ether bridge display higher dopaminergic activity (compared to BBTHIQs with two diaryl ether bridges with one diaryl ether bridge and one biphenyl bridge, or seco derivatives) as exemplified by (1*R*,1′*S*)-thaligrisine (**36**) with IC_50_ values based on the displacement of tritiated raclopride (D_2_-like receptors) and SCH23390 (D_1_-like receptors) of 27 and 5600 nM, respectively.^93^

### Aporphine Alkaloids

Comprising one of the largest groups of natural isoquinolines, aporphine alkaloids are widely distributed in flowering plant families, (e.g., Annonaceae, Aristolochiaceae, Berberidaceae, Canellaceae, Eupomatiaceae, Hernandiaceae, Lauraceae, Leguminosae, Magnoliaceae, Menispermaceae, Monimiaceae, Papaveraceae, Piperaceae, Ranunculaceae, Rhamnaceae, Saururaceae, and Symplocaceae families).^94−97^ The tetracyclic backbone of naturally occurring aporphine alkaloids is typically decorated with substituents such as hydroxyl, methoxy, and methylenedioxy groups on the two aromatic rings. The aporphine alkaloids are a privileged scaffold in drug discovery, as natural aporphine alkaloids are reported to have a myriad of pharmacological activities including antioxidant, antitumor, anticonvulsant, antiplasmodial, antiparkinsonian, antimalarial, antiprotozoal, and cytotoxic effects.^15^

**Figure 9:**
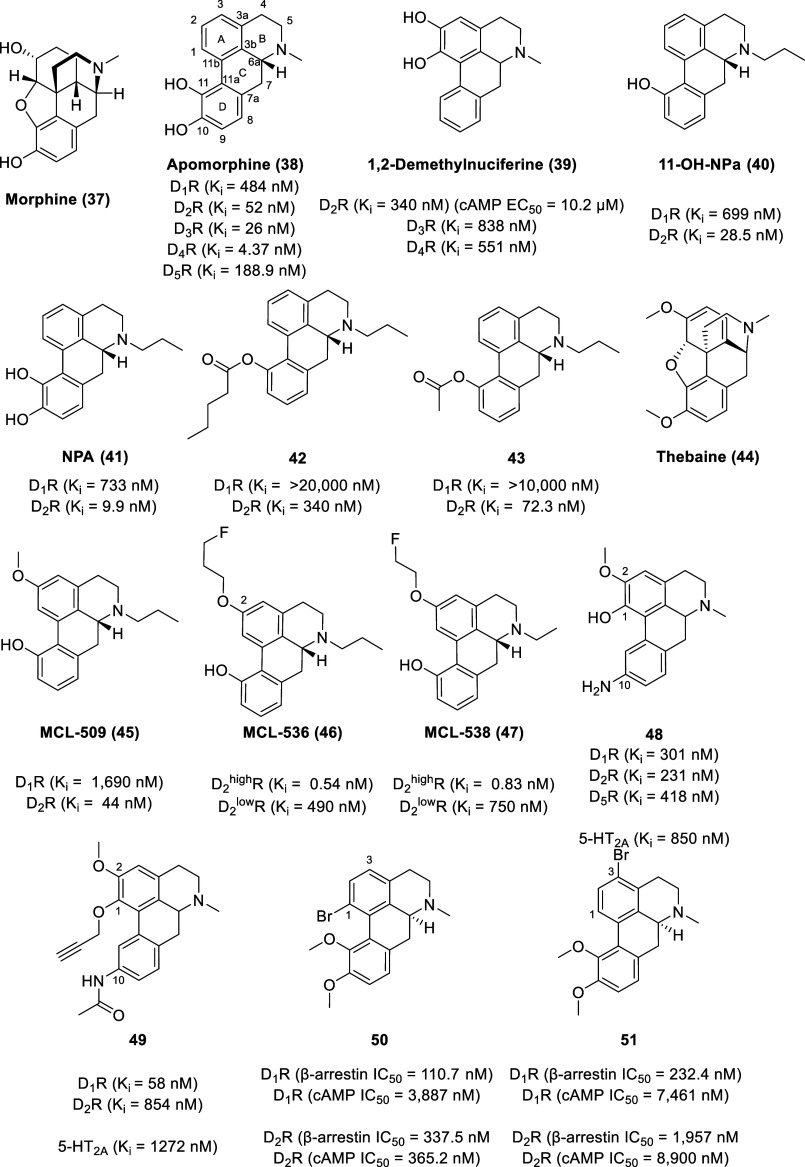
Apomorphine and related chemical structures.

The acid-catalyzed decomposition of morphine (**37**, Figure 9) led to the discovery of the nonselective dopamine receptor partial agonist, apomorphine (**38**) with the affinity trend of D_4_R (*K*_i_ = 4.37 nM) > D_3_R (*K*_i_ = 26 nM) > D_2_R (*K*_i_ = 52 nM) ≫ D_5_R (*K*_i_ = 188.9 nM) > D_1_R (*K*_i_ = 484 nM).^98−100^ Apomorphine is also an antagonist at serotonin receptors (*K*_i_ 5-HT_2A_ = 120 nM, *K*_i_ 5-HT_2B_ = 132 nM, *K*_i_ 5-HT_2C_ = 102 nM) and α-adrenergic receptors (*K*_i_ α_1D_ = 64.6 nM, *K*_i_ α_2A_ = 141 nM, *K*_i_ α_2B_ = 66.1 nM, *K*_i_ α_2C_ = 36.3 nM).^98−100^ Apomorphine was initially explored as an experimental therapeutic for various conditions such as alcoholism, opioid addiction, erectile dysfunction, schizophrenia, and coughing.^101,102^ It is now FDA approved (marketed under the name Apokyn) for the treatment of acute intermittent hypomobility—off episodes with late-stage Parkinson’s disease patients treated with l-DOPA.^103^

By virtue of the *ortho*-catechol ring and phenethylamine moiety, apomorphine shares structural similarity to dopamine (a fundamental rationale for its affinity to dopamine receptors).^104,105^ Due to almost complete first-pass hepatic metabolism (via catechol-*O*-methylation, sulfation, and glucuronidation), apomorphine has very limited oral bioavailability (<4%) and has a short duration of action.^106^ However, apomorphine has the ability to cross the blood–brain barrier freely due to its lipophilic tetracyclic structure. In fact, apomorphine appears to concentrate in the brain, with a brain-to-blood concentration ratio of 8:1.^107^

Apomorphine has undergone extensive SAR studies in efforts to optimize its selectivity, potency, and pharmacokinetic profile. The biphenyl unit, 11-hydroxy substitution, *N*-alkylation, and C-6α (*R*) configuration of aporphines are recognized as essential for dopaminergic activities.^94,95,108^*N*-*n*-Propyl substitution improves D_2_R activity, whereas an *N*-methyl substituent improves D_1_R activity.^109,110^ This propyl effect of improving D_2_R activity can be applied to other dopaminergic molecules.^111^ The presence of a catechol group at the 1,2 position of the aporphine scaffold also favors dopamine receptor affinity, as exemplified by 1,2-demethylnuciferine (**39**).^90^ Despite 1,2-demethylnuciferine displaying submicromolar affinity with the trend of D_2_R (*K*_i_ = 340 nM) > D_4_R (*K*_i_ = 551 nM) > D_3_R (*K*_i_ = 838 nM), 1,2-demethylnuciferine is a weak D_2_R agonist through cAMP signaling with an EC_50_ value of 10.2 μM (*E*_max_ = 50.7% at 10 μM, *E*_max_ = 92.7% at 100 μM) quantified using the HTRF-based cAMP kit in CHO-K1 cells stably expressing the cloned human D_2S_R.

A series of *N*-substituted 11-hydroxynoraporphines and their esters of varying chain lengths was synthesized from morphine and evaluated for binding affinities at dopamine receptor sites in rat caudate-putamen membranes.^112^ From the series, the *N*-*n*-propyl compound **40** (11-OH-NPa) exhibited the highest selectivity and affinity at D_2_R (*K*_i_ D_1_R = 699 nM, *K*_i_ D_2_R = 28.5 nM); however, its D_2_R affinity is lower than that of its catechol precursor **NPA** (**41**, *K*_i_ D_1_R = 733 nM, *K*_i_ D_2_R = 9.9 nM). The valeryl ester of 11-OH-NPa, compound **42**, displayed maximal behavioral potency (via motor activity in normal adult male rats) after systemic injection, whereas the acetate ester of 11-OH-NPa (**43**) showed maximal behavioral potency (via motor activity in normal adult male rats) after enteric (intragastric) administration. Similarly, a series of *N*-alkyl-2-methoxy-11-hydroxynoraporphines was synthesized from thebaine (**44**) and evaluated for binding affinities at dopamine receptors in rat forebrain tissue.^113^ The most selective 11-monohydroxy aporphine was MCL-509 (**45**) (*K*_i_ D_1_ = 1,690 nM, *K*_i_ D_2_ = 44 nM), an orally active Parkinson’s disease drug candidate.^114^

D_2_ receptors exist in two states, either as a functionally inert low-affinity state (D_2_^low^R) or as a functional high-affinity state (D_2_^high^R).^115^ The high-affinity and low-affinity receptors show a biphasic competition curve and have different binding affinities to various ligands. Evidence increasingly indicates that alterations in the density of D_2_ receptors in the high-affinity state are more important to pathophysiological processes than alterations in the total receptor density. As such, the development of a selective D_2_R agonist that is capable of distinguishing between the high- and the low-affinity states is critical as a tool for diagnostics and therapeutics for psychosis and Parkinson’s disease.^115^ Fluoroalkylation of a hydroxyaporphine substrate led to the development of MCL-536 (**46**; *K*_i_ D_2_^high^R = 0.54 nM, *K*_i_ D_2_^low^R = 490 nM) and MCL-538 (**47**; *K*_i_ D_2_^high^R = 0.83 nM, *K*_i_ D_2_^low^R = 750 nM) as novel D_2_^high^R-targeted probes for the dopaminergic system.^116−118^

Due to the high sequence identity (82% in transmembrane regions) between D_1_R and D_5_R, the development of subtype-selective D_1-like_ ligands is challenging and limited to only a few compounds.^7,119−121^ The development of ligands with D_1_R versus D_5_R selectivity (and vice versa) remains an area of significant interest in the field. In 2020, our group reported the synthesis and evaluation of two series of aporphine analogs bearing either a sole C-10 nitrogen substituent on the tetracyclic aporphine core or 1,2,10-trisubstituted aporphines (with both groups containing a nitro, aniline, or amide moiety at the C-10 position).^122^ All compounds except compound **48** lacked D_5_R affinity. The C-10 nitrogen monosubstituted analogs exhibited a preference for the serotonin 5-HT_1A_R, while the 1,2,10-trisubstituted analogs generally displayed dopamine receptor selectivity. Out of the two series of compounds, the 1,2,10-trisubstituted aporphine **49** was identified as the ligand with the highest affinity toward D_1_R (*K*_i_ = 58 nM) with low affinity toward 5-HT_2A_R (*K*_i_ = 1272 nM) and D_2_R (*K*_i_ = 854 nM) and no affinity for the other receptors tested (5-HT_1A_R and D_5_R). Compound **49** displays higher metabolic stability than apomorphine in human liver microsomes, which is not surprising as it lacks any common conjugation sites (amino, carboxy, hydroxy, or thiol groups) for phase II metabolism. Computational simulations revealed that the D_1_R versus D_5_R selectivity of **49** might arise from stronger hydrophobic contacts (primarily with phenylalanine residues) with D_1_R compared to D_5_R.

With the potential to bypass observed adverse effects, functional selectivity enables the activation of distinct subsequent signaling cascades or even the selective activation of a particular G protein subtype.^22,123^ As functional selectivity is still a relatively new paradigm in drug discovery, very few such studies have been performed on aporphines. A structure–functional–selectivity relationship (SFSR) study of the apomorphine scaffold revealed the structural motifs responsible for biased activity at both D_1-like_ and D_2-like_ receptors.^124^ It was found that alkyl and halogen substituents on the catechol ring generally do not contribute to functional selectivity; rather, these substituents reduce agonist activity at dopamine receptors for both β-arrestin 2 and G protein pathways. β-Arrestin-biased antagonism at both D_1_ and D_2_ receptors was observed with bromo substitution at either the C-1 (**50**) [D_1_R (β-arrestin IC_50_ = 110.7 nM, cAMP IC_50_ = 3,887 nM), D_2_R (β-arrestin IC_50_ = 337.5 nM, cAMP IC_50_ = 365.2 nM)] or the C-3 (**51**) [D_1_R (β-arrestin IC_50_ = 232.4 nM, cAMP IC_50_ = 7,461 nM), D_2_R (β-arrestin IC_50_ = 1,957 nM, cAMP IC_50_ = 8,900 nM)] position measured by cAMP GloSensor and β-arrestin bioluminiscence resonance energy transfer (BRET) assays. These compounds may be useful for further hit-to-lead optimizations, particularly for the treatment of hyperdopaminergia, which is implicated in psychiatric disorders such as ADHD and schizophrenia.

**Figure 10:**
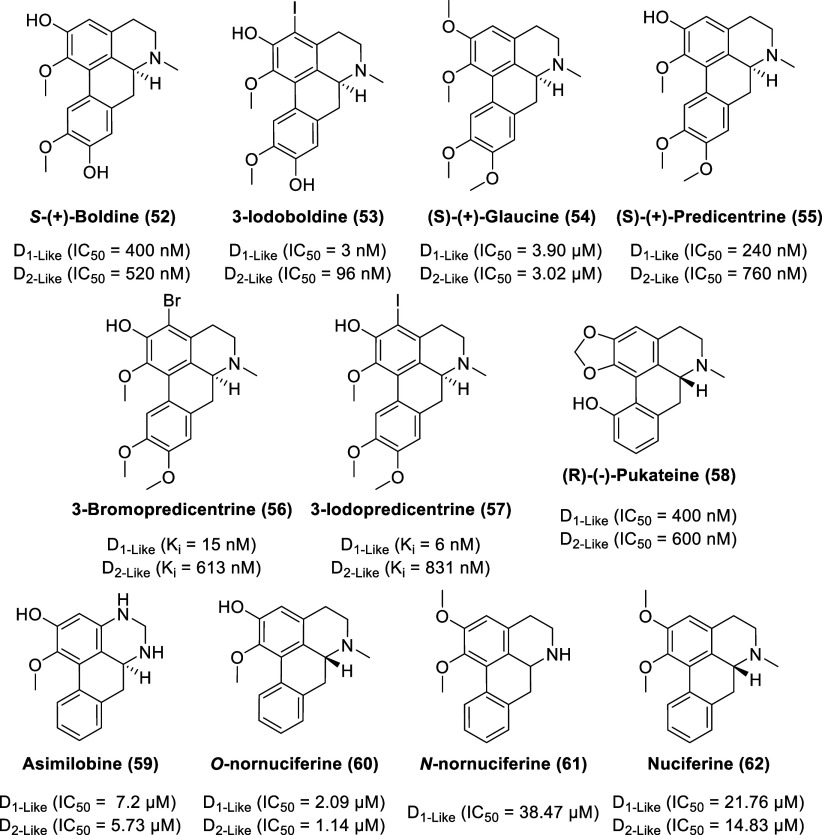
Selected aporphine alkaloids and related chemical structures.

The natural aporphine, *S*-(+)-boldine (**52**, Figure 10), well known as a constituent of the bark and leaves of the Chilean tree Boldo (*Peumus boldus* Molina, Monimiaceae),^125−127^ has served as a template for the construction of new aporphines. Boldo is used in folk medicine, primarily for the treatment of liver ailments. Although boldine is recognized as an effective antioxidant, it also displays antagonist activity at D_1-like_ and D_2-like_ receptors with IC_50_ values of 400 and 520 nM, respectively, based on the displacement of [^3^H]-SCH 23390 or [3H]-raclopride from their specific binding sites in rat striatum.^128^ Despite the affinity toward dopamine receptors, boldine does not display effective central dopamine antagonist activities in vivo as the high dose of 40 mg/kg boldine i.p. failed to produce any effect on striatal [^3^H]raclopride binding in rat forebrain while [^3^H]-SCH 23390 binding decreased by 25%. Furthermore, orally administered boldine is rapidly glucuronidated in the liver with a plasma elimination half-life of 31 min.^129^ Molecular bromine in acetic acid or *N*-halosuccinimides in trifluoroacetic acid was used to halogenate boldine to afford 3-haloboldines and 3,8-dihaloboldines.^130^ Halogenation of boldine at C-3 favors affinity for rat brain D_1-like_ dopamine receptors compared to D_2-like_ dopamine receptors, as exemplified by 3-iodoboldine (**53**) with IC_50_ values of 3 (D_1-like_ receptors) and 96 nM (D_2-like_ receptors) based on radioligand displacement studies in rat-brain membranes.

(*S*)-(+)-Glaucine (**54**) is a natural derivative of boldine, with no free hydroxyl group, and is used as an antitussive agent (mainly due to Ca^2+^ channel antagonism). It is commercially isolated from the yellow horned poppy (*Glaucium flavum* Crantz).^131^ Glaucine displays a lower affinity toward dopamine receptors than boldine, with IC_50_ values of 3.90 (D_1-like_ receptors) and 3.02 μM (D_2-like_ receptors), measured by binding studies in rat-striatal membranes using [^3^H]-SCH 23390 or [^3^H]-raclopride.^128^ In vivo studies reveal some antidopaminergic properties of glaucine as the high dose of 40 mg/kg glaucine i.p. elicited a reduction of 50% for both [^3^H]-SCH 23390 and [^3^H]-raclopride binding in rat forebrain. Both boldine and glaucine inhibited penile erection, and apomorphine (0.1 mg/kg s.c.) induced yawning in rat models by over 50% at 40 mg/kg (i.p.) without impacting the metabolism of dopamine in rat and mouse forebrain tissues. Similar to boldine and glaucine, (*S*)-(+)-predicentrine (**55**) displays moderate activity at both D_1-like_ and D_2-like_ receptors with IC_50_ values of 240 (D_1-like_ receptors) and 760 nM (D_2-like_ receptors) based on radioligand displacement studies in rat-striatal membranes.^132^ 3-Halogenated and 3,8-dihalogenated derivatives of predicentrine had improved affinity for D_1-like_ receptors, as demonstrated by 3-bromopredicentrine (**56**; *K*_i_ D_1-like_ = 15 nM, *K*_i_ D_2-like_ = 613 nM) and 3-iodopredicentrine (**57**; *K*_i_ D_1-like_ = 6 nM, *K*_i_ D_2-like_ = 831 nM).^132^ Halogenated glaucine derivatives failed to exhibit any clear trend toward enhanced selectivity or any improvement with potency, indicating that the D_1-like_ enhanced potency with halogenation at C-3 is conditional with the presence of the 2-hydroxy group on the aporphine skeleton (and it is this moiety that predominantly determines the binding mode that favors D_1-like_ over D_2-like_ receptors).

As an isolate from the bark of the pukatea tree (*Laurelia novae-zelandiae*), (*R*)-(−)-pukateine (**58**) is another aporphine congener with antioxidant and dopaminergic properties.^133^ The monohydroxyaporphine derivative displays IC_50_ values of 400 (at D_1-like_ receptors) and 600 nM (at D_2-like_ receptors) based on radioligand binding experiments. Pukateine bears a meta hydroxyphenyl (at C-11) and 6a*R* configuration, both of which are associated with dopamine receptor activation.^94,134^ In 6-hydroxydopamine unilaterally denervated rats, a dose of 8 mg/kg but not 4 mg/kg of pukateine elicited a significant contralateral circling, which is a behavior classically associated with the actions of a dopamine agonist. Perfusion of pukateine (at 340 μM) through a microdialysis probe inserted into the striatum induced a significant increase in dopamine levels, indicating an agonist-like interaction with dopamine receptors.

Extracted from the leaves of the sacred lotus *Nelumbo nucifera* Gaertn., four bioactive aporphine alkaloids [asimilobine (**59**), *O*-nornuciferine (**60**), *N*-nornuciferine (**61**), and nuciferine (**62**)], were identified as antagonists with IC_50_ values in the mid- to low-micromolar range for dopamine receptors.^135^*O*-Nornuciferine exhibited the highest potency at both D_1_R (IC_50_ = 2.09 μM) and D_2_R (IC_50_ = 1.14 μM), using FLIPR assays in HEK293 cell lines.

### Cularine Alkaloids

**Figure 11:**
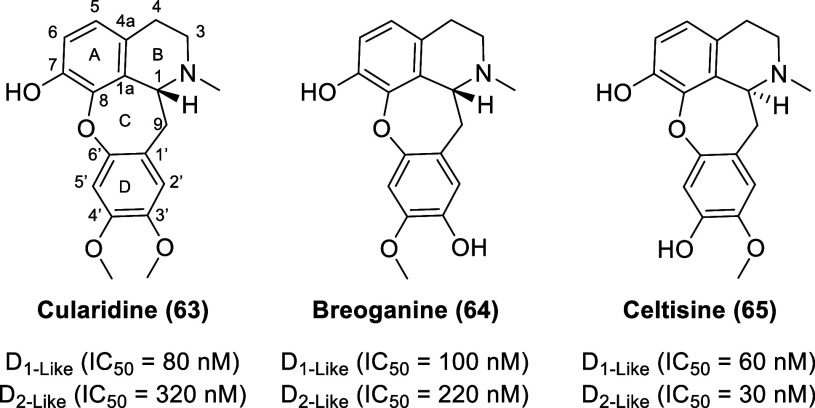
Chemical structures of cularidine, breoganine, and celtisine.

Isolated from the aerial parts of the Spanish shrub *Sarcocapnos crassifolia* (Fumariaceae), the natural cularines—cularidine (**63**, Figure 11), breoganine (**64**), and celtisine (**65**)—display dopamine receptor affinity.^136^ The cularines bear structural resemblance to aporphines but have a tetracyclic skeleton featuring a 7-membered oxepine system rather than the 6-membered carbocyclic ring C moiety found in aporphines. Cularines and aporphines are biosynthetically derived from similar benzylisoquinoline precursors.^137^ Cularidine displays IC_50_ values of 80 (D_1-like_ receptors) and 320 nM (D_2-like_ receptors) (0.25-fold selectivity for D_l_R versus D_2_R), whereas breoganine displays IC_50_ valuesof 100 (D_1-like_ receptors) and 220 nM (D_2-like_ receptors) (0.45-fold selectivity for D_l_R versus D_2_R) derived from radioligand displacement studies in rat-striatal membranes. Celtisine displays IC_50_ values of 60 (D_1-like_ receptors) and 30 nM (D_2-like_ receptors) (2-fold selectivity for D_l_R/D_2_R). To date, there have been no follow-up studies to explore the potential of cularine derivatives as dopamine receptor ligands.

### Phenanthrene Alkaloids

**Figure 12:**
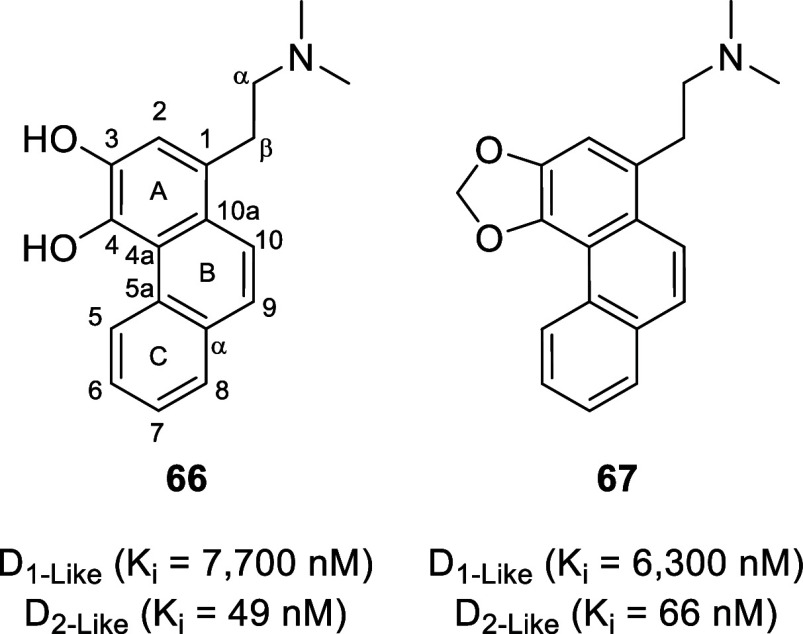
Chemical structures of 3,4-dihydroxy- and 3,4-methylenedioxyphenanthrene-type alkaloids.

While phenanthrene alkaloids do not contain a nitrogen heterocycle, they are considered alkaloids due to being biogenetically derived from aporphines (thus occurring in the same plant families as aporphines).^138^ The degradation of aporphines (for the most part) readily yields phenanthrene alkaloids. Hoffmann degradation is the classical (synthetic) method for the transformation of aporphines into phenanthrene alkaloids. The reaction involves the formation of a quaternary ammonium salt by treatment with an alkylating agent (usually methyl iodide or dimethyl sulfate) followed by anion exchange with silver oxide to form a quaternary ammonium hydroxide which can then be thermolyzed to the phenanthrene. 3,4-Dihydroxy- and 3,4-methylenedioxyphenanthrenic derivatives (**66** and **67**, respectively, Figure 12) displayed high selectivity toward D_2-like_ receptors.^139^**66** displays affinity values (*K*_i_) of 7700 nM at D_1-like_ receptors and 49 nM at D_2-like_ receptors (*K*_i_ D_1_/D_2_ = 157), whereas **67** displays affinity values of 6300 nM at D_1-like_ receptors and 66 nM at D_2-like_ receptors (*K*_i_ D_1_/D_2_ = 96).

### Berberine and Tetrahydroprotoberberines (THPBs)

**Figure 13:**
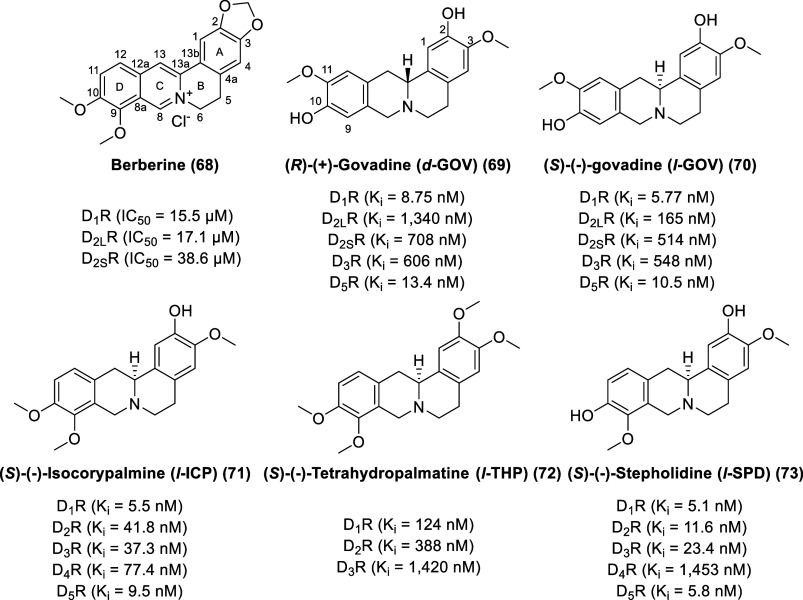
Chemical structures of berberine and selected tetrahydroprotoberberines.

The bright yellow compound berberine (**68**, Figure 13), a characteristic isolate of most barberries (*Berberis* plants), of the American goldenseal *Hydrastis canadensis*, and of the Chinese herb *Coptis chinensis*, is traditionally used as a textile dye and antiparasitic agent by many cultures in Eurasia and the Americas.^140,141^ Berberine displays a wide variety of pharmacological properties with implications for cancer, digestive diseases, neurological diseases, cardiovascular diseases, and metabolism disorders. Berberine is a weak dopamine D_1-like_ and D_2-like_ receptor antagonist (D_1_R IC_50_ = 15.5 μM, D_2L_R IC_50_ = 17.1 μM, D_2S_R IC_50_ = 38.6 μM) (using HTRF assay for D_1_R and [^35^S]GTPγS binding assay for D_2S_R and D_2L_R) and ameliorates the development of experimentally induced colitis in mice by suppressing innate and adaptive immune responses.^142^

Usually isolated from *Corydalis* and *Stephania* species, tetrahydroprotoberberines (THPBs) have been found to display profound effects on the dopaminergic system.^143,144^ Several THPB natural products and their synthetic derivatives have been identified as dopamine receptor ligands with affinities and activities being primarily reported at D_1_R, D_2_R and (to a lesser extent) D_3_R. In THPB natural products, as in all of the biosynthetically related isoquinoline alkaloids, the aryl rings of the tetracyclic nucleus are usually substituted with hydroxyl or methoxyl groups in the two aryl rings. With a clear consensus, behavioral and biochemical evidence has illustrated that THPBs are antagonists at the D_2_ family of dopamine receptors.^145^ The intrinsic activity of THPBs at the D_1_ family of dopamine receptors is variable, with a majority of compounds being reported as antagonists and comparatively fewer as partial agonists.

Enantiomers of govadine exhibited drastically different behavioral and pharmacological properties.^146^ (*R*)-(+)-Govadine (d-GOV, **69**) displays dopamine receptor binding affinity with the order of D_1_R (*K*_i_ = 8.75 nM) > D_5_R (*K*_i_ = 13.4 nM) ≫ D_3_R (*K*_i_ = 606 nM) > D_2S_R (*K*_i_ = 708 nM) ≫ D_2L_R (*K*_i_ = 1,340 nM), whereas (*S*)-(−)-govadine (l-GOV, **70**) displays dopamine receptor binding affinity with the trend of D_1_R (*K*_i_ = 5.77 nM) > D_5_R (*K*_i_ = 10.5 nM) ≫ D_2L_R (*K*_i_ = 165 nM) > D_2S_R (*K*_i_ = 514 nM) > D_3_R (*K*_i_ = 548 nM). d-GOV and l-GOV exhibit modest affinity for adrenergic receptors [d-GOV (K_i_ α_1A_ = 648 nM, *K*_i_ α_1B_ = 2,590 nM, *K*_i_ α_1D_ = 263 nM, *K*_i_ α_2A_ = 304 nM), l-GOV (*K*_i_ α_1A_ = 369 nM, *K*_i_ α_1B_ = 2480 nM, *K*_i_ α_1D_ = 235 nM, *K*_i_ α_2A_ = 223 nM)]. Rodent models predictive of antipsychotic efficacy and of positive symptoms of schizophrenia showed that l-GOV (but not d-GOV) had improved these measures. On the other hand, d-GOV (but not l-GOV) may be an effective cognitive enhancer, as it improved impairments in compromised memory function in delayed response tasks.

Isolated from the plant *Corydalis yanhusuo*, (*S*)-(−)-isocorypalmine (l-ICP, **71**) exhibits strong dopamine receptor binding affinity with the order of D_1_R (*K*_i_ = 5.5 nM) > D_5_R (*K*_i_ = 9.5 nM) > D_3_R (*K*_i_ = 37.3 nM) > D_2_R (*K*_i_ = 41.8 nM) > D_4_R (*K*_i_ = 77.4 nM).^147,148^l-ICP is a partial agonist at D_1-like_ receptors and antagonist at D_2-like_ receptors.

(*S*)-(−)-Tetrahydropalmatine (l-THP, **72**) displays modest nanomolar to micromolar affinity and antagonist activity toward dopamine receptors with the order of D_1_R (*K*_i_ = 124 nM) > D_2_R (*K*_i_ = 388 nM) ≫ D_3_R (*K*_i_ = 1420 nM).^145,149^ The *levo* isomer of THP is responsible for the analgesic, sedative, and neuroleptic properties, whereas the *dextro* (*R*) isomer of THP has a distinct toxicologic action.^143,150^l-THP has been approved for use as an analgesic and sedative in China for over 50 years, originally isolated from *Stephania rotunda*, under the brand Rotundine or Rotundin.

(*S*)-(−)-Stepholidine (l-SPD, **73**), a constituent of *Stephania spp*, displays higher dopamine receptor affinity than l-THP, with the trend of D_1_R (*K*_i_ = 5.1 nM) > D_5_R (*K*_i_ = 5.8 nM) > D_2_R (*K*_i_ = 11.6 nM) > D_3_R (*K*_i_ = 23.4 nM) ≫ D_4_R (*K*_i_ = 1453 nM).^151^l-SPD was originally reported to exhibit sedative and analgesic effects on the CNS and to decrease blood pressure without adverse cardiac effects.^152−154^ Its antipsychotic potential has also been investigated due to its reported mixed functional profile at D_1_R and D_2_R (D_1_R agonist/D_2_R antagonist).^155−158^

Substance use disorder is defined as a compulsive and habitual nonmedical self-administration of drugs despite having severe adverse consequences.^159^ Addictive drugs elevate extracellular levels of dopamine, rewiring the brain’s dopaminergic mechanisms.^160^ These alterations in the dopaminergic pathways result in decreased dopamine receptor expression in the brain, reducing interest in all activities but those that arise from habitual rewards. Due to the dopamine receptor polypharmacology of THPBs, these compounds have been proposed to be a novel source of pharmacotherapies for substance use disorders.^145,149^ In animal models of substance misuse, l-THP demonstrated attenuation of self-administration of cocaine, methamphetamine, heroin, and nicotine.^161−167^l-ICP was also shown to reduce behavioral sensitization and rewarding effects of cocaine in mice.^147^ Likewise, l-SPD displayed attenuation of reinstatement of drug and cue-induced self-administration of both opiates and psychostimulants.^168−171^

The clinical evidence for the efficacy of THPBs in substance use disorders is limited only to a single preliminary clinical trial conducted in China, examining the effects of l-THP on ameliorating heroin craving and increasing the abstinence rate in heroin users via a randomized, double-blinded, and placebo-controlled study of 120 heroin-dependent patients.^172^ The results suggest that l-THP has presumably therapeutic utility in the treatment of heroin addiction as the drug significantly ameliorated protracted abstinence withdrawal syndrome (PAWS). This pilot study does present several methodological limitations including not separating treatment seekers and nontreatment seekers (as they may have different responses to l-THP treatment), employing only pharmacotherapy without cognitive behavioral treatment (CBT), and utilizing only one low dose of l-THP with a short duration of 1 month to limit the risk of possible hepatic toxicity. Additional clinical evidence revealed that short-term l-THP administration does not exhibit significant differences in vital signs or side effects compared to the placebo group in cocaine users.^173^

THPBs can be semisynthetically prepared by the reduction of the relatively abundant protoberberines such as berberine, palmatine, and their partially *O*-demethylated analogs or synthetically prepared via Pictet–Spengler reaction, Bischler–Napieralski reaction, Pomeranz–Fritsch reaction, or Mannich reaction to construct the B-ring and C-ring in sequence.^174^

**Figure 14:**
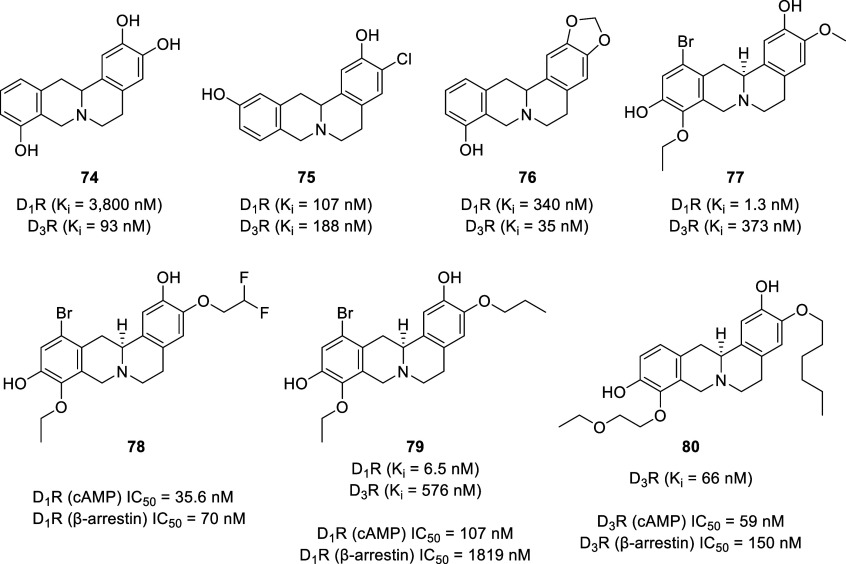
Chemical structure of selected synthetic THPB analogs.

Initial studies on synthetic tetrahydroprotoberberines, focusing on 2,3,9- and 2,3,11-trisubstitution patterns, revealed several ligands with dopaminergic activity.^175^ The 2,3,9-trihydroxy-THPB (**74**, Figure 14) exhibited the highest selectivity for the D_2-like_ receptors (*K*_i_ D_1_/D_2_ = 41) with affinity values (*K*_i_) of 3800 nM at D_1-like_ receptors and 93 nM at D_2-like_ receptors. Replacement of the 3-OH with a chlorine atom on the A-ring (3-Cl-2,11-diOH, **75**) resulted in increased affinity for D_1-like_ receptors (*K*_i_ = 107 nM) but dramatically reduced the selectivity (D_2-like_ receptors, *K*_i_ = 188 nM) (*K*_i_ D_1_/D_2_ = 0.6), which is in contrast to previous studies on BTHIQs where chlorination ortho to the oxygenated group in the A-ring resulted in increased affinity and selectivity toward D_2-like_ receptors.^85,176,177^ The presence of a methylenedioxy group in the 2,3 position (2,3-OCH_2_O-9-OH, **76**) exerted lower selectivity for the D_2-like_ receptors (*K*_i_ D_1_/D_2_ = 10) with improved affinity for both dopamine types (D_1-like_ receptors, *K*_i_ = 340 nM; D_2-like_ receptors, *K*_i_ = 35 nM).

With a lower risk of drug–drug interactions compared to multicomponent drugs (drug cocktails), a designed multiple ligand is a single chemical entity that can modulate multiple targets simultaneously, thereby enhancing efficacy and optimizing dosing and safety.^178−180^ Dual-acting ligands may be similar to bivalent ligands as they may also combine two pharmacophoric units linked by a spacer, except the spacer is shorter than those of bivalent ligands (with the aim not to simultaneously bind to both protomers but rather to simply deliver both pharmacophoric moieties simultaneously).^178,181^ Due to their inherent pharmacological properties, bivalent ligands tend to be better suited as pharmacological tools whereas dual-acting ligands may have further application as therapeutic agents.^179^

In efforts to improve the efficacy and safety of THPBs, our group is actively working on fine tuning the polypharmacology of THPBs as selective D_1_R partial agonist/D_3_R antagonist dual-acting ligands.^182−185^ This targeting strategy is based on the observation that coadministration of a D_1_R partial agonist (SKF 77434) and a D_3_R antagonist (NGB 2904) produced robust decreases in cocaine seeking and reward in rats. This indicates a synergistic effect between D_1_R agonism and D_3_R antagonism.^186^ Our initial efforts were focused on SAR studies with l-SPD as a lead molecule. So far, our attempts to identify new selective D_1_R partial agonist/D_3_R antagonist dual-acting ligands from THPBs have not been successful, although we have identified several dopamine receptor subtype-specific ligands (e.g., **77**–**80**).^182−185,187^ Our structure–affinity relationship studies on l-SPD revealed that bromination at the C-12 position improved D_1_R affinity but generally diminished D_3_R affinity. Homologation at the C-9 position improved D_1_R selectivity versus D_2_R and D_3_R (often lacking affinity at D_2_R), whereas alkylation of the C-10 phenolic group with up to 6 carbon atoms in length was tolerated for D_1_R affinity. Homologation at the C-3 position generally led to retention of the magnitude of D_3_R affinities with the notable exception of fluoroethylation, which resulted in an approximate 3-fold reduction in D_3_R affinity compared to l-SPD.

**Figure 15:**
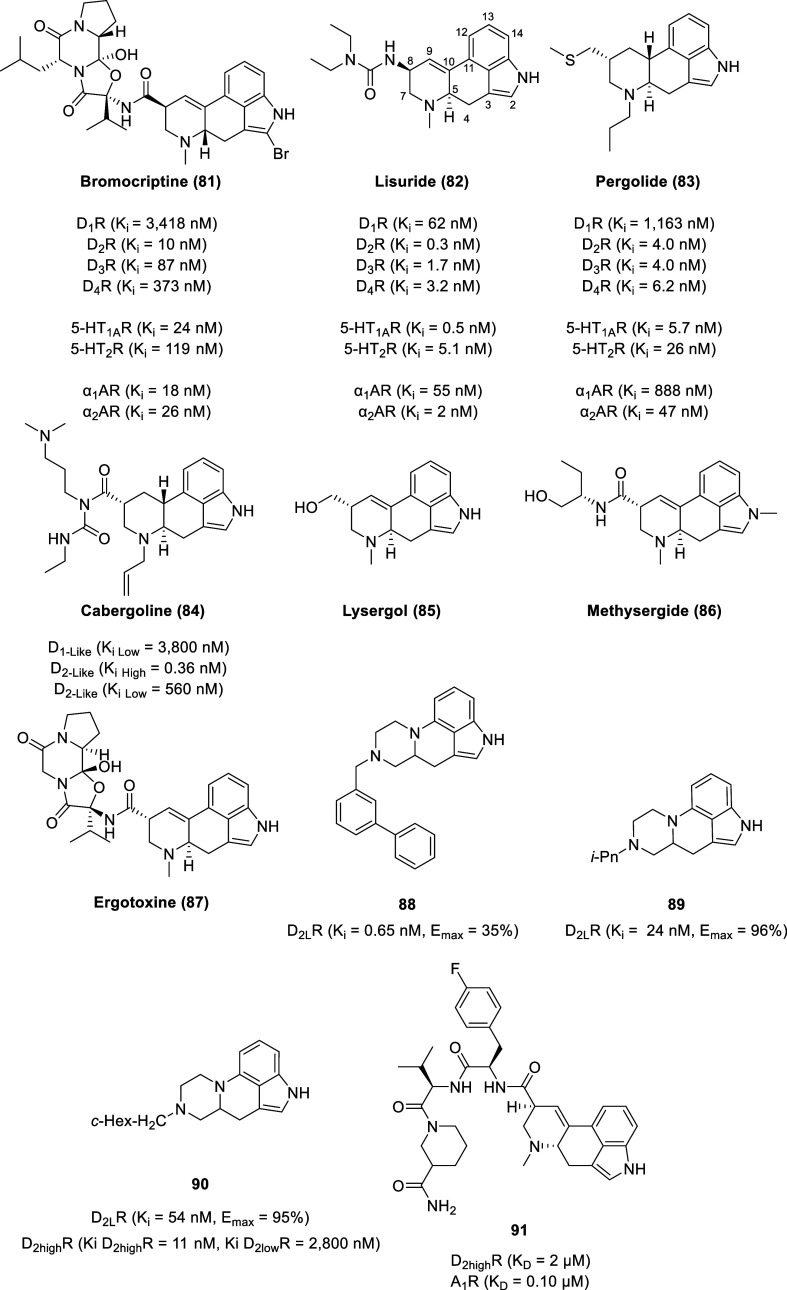
Selected ergolines and related chemical structures.

Only a handful of studies have been undertaken to examine the functional selectivity bias of THPBs. Data gathered to date by our group suggest that THPBs function as antagonists at both G protein and arrestin pathways (Figure 15) (cAMP functional assays, Promega’s split luciferase-based GloSensor cAMP biosensor technology, or Lance Ultra cAMP IC50 assays; β-arrestin assays, TANGO assays, or DiscoveRx PathHunter technology). l-SPD was found to be a potent pan-dopamine receptor antagonist of both G protein- and β-arrestin-mediated signaling (thus lacking functional selectivity), determined using DiscoveRx PathHunter technology.^151^

## 4 Ergolines

First identified in the dark dense sclerotia of infected grains and grasses by parasitic fungi of the genus *Claviceps*, ergot alkaloids are also produced by a wide variety of other filamentous fungi, including species in the genus *Arthroderma*, *Aspergillus*, *Balansia*, *Epichloe*, *Neotyphodium*, *Penicillium*, and *Periglandula*.^188−193^ Embedded within the tetracyclic structure of the ergolines are the defining features of the monoamine neurotransmitters serotonin, noradrenaline, and dopamine, empowering ergolines to act as potent agonists, partial agonists, or antagonists at these receptors.^194^ Derivatives of ergolines are categorized into 3 main classes: lysergic acid and its simple amides, ergopeptines, and clavines.^195,196^ Lysergic acid derivatives contain a carboxylic acid group at position 17 with most lysergic acid derivatives being functionalized by an amide linkage to that position (e.g., ergine and ergonovine). The peptide ergot alkaloids are defined by a tripeptide-derived, cyclol-lactam structure attached as a C-17 amide substituent (e.g., ergocristine, ergotamine, and ergovaline). The clavine alkaloids are classified as the derivatives of 6,8-dimethylergoline or the tricyclic precursors (i.e., agroclavine, elymoclavine, and festuclavine).

While naturally occurring ergolines generally lack selectivity between dopamine receptor subtypes and other biogenic amine receptors, a select number of semisynthetic ergolines display a preference toward dopamine receptors.^194^ Several ergolines are used as antiparkinsonian drugs [e.g., bromocriptine (**81**), lisuride (**82**), and pergolide (**83**), Figure 15] and inhibitors of prolactin release [e.g., **81**–**83** and cabergoline (**84**)] due to their agonist activity at dopamine receptors.^197,198^ The simulation of neostriatal D_1_ and D_2_ receptors is associated with the antiparkinsonian effect of ergolines, whereas the simulation of D_2_ or D_4_ receptors mediates the suppression of prolactin secretion from the anterior pituitary.^199,200^

Reduction of the methyl ester of lysergic acid with lithium aluminum hydride yields lysergol (**85**), a key intermediate in the preparation of semisynthetic ergoline analogs.^201^ As the hydroxy group of lysergol could undergo nucleophilic substitution upon conversion to sulfonic ester (e.g., mesylate or tosylate), researchers at Lilly were able to introduce a methylthio moiety leading to pergolide.^202^ Zikán and Semonský at the Research Institute for Pharmacy and Biochemistry at Prague (later SPOFA) developed lisuride via derivation of methysergide (**86**) in efforts toward the development of an antimigraine agent.^203^ In 1954, Moses Shelesnyak recognized that ergotoxine (**87**) could inhibit deciduoma formation that was associated with ovum implantation in rats, attributable to inhibition of prolactin secretion.^201,204^ This led to researchers at Sandoz developing ergot analogs that selectively inhibited prolactin secretion, leading to the development of bromocriptine.^201,205^

Distal substituents on an ergoline-derived scaffold by combining two privileged scaffolds, indole and phenylpiperazine (**88**–**90**), yielded potent D_2_R ligands with intrinsic activities ranging from full agonism to partial agonism.^206^ Alkylation on the nitrogen on the piperazine moiety led to a series of D_2_R agonists with a wide range of binding affinities with the most potent D_2L_R ligand bearing an *N*-(1,1′-biphenyl-3-yl)methyl group (**88**) (*K*_i_ = 0.65 nM, *E*_max_ = 35%). An inverse relationship was observed between the size of the amine substituent and the D_2_R efficacy, where smaller alkyl substituents are full agonists (e.g., **89** and **90**). Alkylation of the nitrogen on the indole moiety resulted in ligands with serotonin 5-HT_6_ receptor affinity.

On both a functional and a molecular level, adenosine and dopamine receptors have interactions with each other as heteromeric complexes.^207^ Adenosine receptor-selective antagonists display antiparkinsonian activities and exhibit neuroprotective properties (rarely seen with currently used antiparkinsonian drugs). As such, combining adenosine A1 receptor (A_1_R) and A_2A_R antagonism with dopamine D_1_R and D_2_R agonism into a single multivalent ligand could lead to novel treatments for Parkinson’s disease with improved efficacy and safety.^208,209^ As ergot alkaloids are privileged structures with an inherent ability to interact with diverse biological receptors, a combinatorial approach based on the ergoline scaffold allowed the identification of compounds with dual adenosine antagonist and dopamine agonist activity as exemplified by **91** (A_1_R *K*_D_ = 0.10 μM, D_2_^high^R *K*_D_ = 2 μM).^210^

## 5 Miscellaneous Dopamine Receptor-Targeted Natural Products

**Figure 16:**
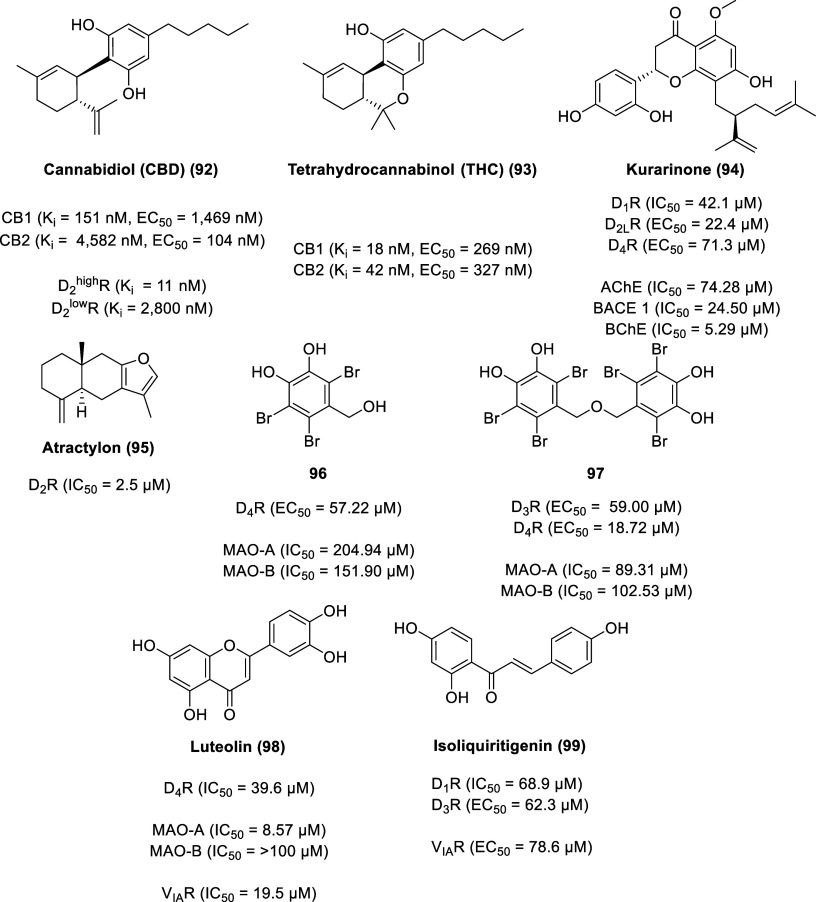
Miscellaneous dopamine receptor-targeted natural products.

Cannabidiol (CBD, **92**, Figure 16) is a phytocannabinoid found in the *Cannabis sativa* plant, accounting for up to 40% of the plant’s extract.^211^ Compared to Δ^9^-tetrahydrocannabinol (Δ^9^-THC, **93**), CBD lacks psychotomimetic and other psychotropic effects. Δ^9^-THC displayed binding affinity and functional activity (using a GTPγS functional bioassay) in the nanomolar range for both cannabinoid receptors [(*K*_i_ (CB1) = 18 nM, *K*_i_ (CB2) = 42 nM)] [(EC_50_ (CB1) = 269 nM, EC_50_ (CB2) = 327 nM)].^212^ With the exception of functional activity at CB2, CBD displays lower binding affinity and functional activity than Δ^9^-THC at the cannabinoid receptors [(*K*_i_ (CB1) = 151 nM, *K*_i_ (CB2) = 4582 nM)] [(EC_50_ (CB1) = 1469 nM, EC_50_ (CB2) = 104 nM)].

CBD is FDA approved for the treatment of seizures associated with Lennox–Gastaut syndrome, Dravet syndrome, and tuberous sclerosis complex in patients 1 year of age and older (under the brand name Epidiolex). CBD is implicated in other medical illnesses including anxiety, psychosis, and depression, although it is not FDA approved for these conditions.^211,213^ The clinical antipsychotic effects of CBD correlate with its partial agonist activity at D_2_^high^R (*K*_i_ D_2_^high^R = 11 nM, *K*_i_ D_2_^low^R = 2800 nM) in a similar biphasic manner as aripiprazole, a dopamine partial agonist antipsychotic drug.^214^

Kurarinone (**94**) is an abundant lavandulylated flavonoid isolated from the perennial shrub *Sophora flavescens* (Fabaceae).^215^ Known as Kushen, the dried roots of *S. flavescens* are used in traditional Chinese medicine for the treatment of cancer and inflammatory diseases.^216^ Kurarinone displays moderate to potent inhibitory activity on acetylcholinesterase (AChE, IC_50_ = 74.28 μM), beta-secretase 1 (BACE 1, IC_50_ = 24.50 μM), and butyrylcholinesterase (BChE, IC_50_ = 5.29 μM) using either Ellman’s colorimetric method (for AChE and BChE) or with BACE1 FRET assay kit (β-Secretase) kit.^217,218^ Functional HTRF-based cAMP assays (for D_1_R and D_4_R) and Ca^2+^ flux assays (for D_2L_R) revealed kurarinone exhibited an antagonist effect on D_1_R (IC_50_ = 42.1 μM) and an agonist effect on D_2L_R (EC_50_ = 22.4 μM) and D_4_R (EC_50_ = 71.3 μM), suggesting its potential to alleviate various neurodegenerative diseases (NDDs).^215^

Isolated from the perennial herb *Atractylodes macrocephala* Koidz, atractylon (**95**) is a furan-containing compound with several pharmacological activities, including anticancer, antiviral, anti-inflammation, and gastroprotective properties.^219^ Recently, it was demonstrated that atractylon is a D_2_R agonist in a dose-dependent manner (using a piggyBac-TANGO assay) with an IC_50_ of 2.5 μM.^220^ Atractylon attenuated motor deficits and abnormal gait disturbances as well as protected dopaminergic neurons in MPTP-induced PD mice, indicating potential as a therapeutic agent in the treatment of motor symptoms of Parkinson’s disease.

Monoamine oxidase (MAO) is a mammalian flavoenzyme that catalyzes the inactivation pathway of several neurotransmitters (e.g., adrenaline, dopamine, norepinephrine, and serotonin) through oxidative deamination.^221^ In the biochemical process, oxidative damage can occur as hydrogen peroxide is the major and direct side product of MAO activity. Thus, inhibitors of MAO have neuroprotective properties.^222^ MAO exists in two isoforms, MAO-A and MAO-B, with different tissue distribution and substrate-inhibitor recognition sites. Bromophenols (**96** and **97**) from the red alga *Symphyocladia latiuscula* are good D_3_R/D_4_R agonists and are moderate MAO inhibitors and as such are implicated in the management of neurodegenerative diseases.^223^ Compound **96** displayed inhibition of MAO (MAO-A IC_50_ = 204.94 μM, MAO-B IC_50_ = 151.90 μM) and had a potent agonist effect with D_4_R (EC_50_ = 57.22 μM), whereas **97** displayed inhibition of MAO (MAO-A IC_50_ = 89.31 μM, MAO-B IC_50_ = 102.53 μM) and had an agonist effect with both D_4_R (EC_50_ = 18.72 μM) and D_3_R (EC_50_ = 59.00 μM) (calculated from a log dose–inhibition curve or concentration-dependent agonist response curve).

Luteolin (**98**) is another flavonoid, widely distributed in the plant kingdom (e.g., *Artemisia montana*, *Cirsium japonicum*, *Cynara scolymus*, and *Taraxacum officinale*).^224^ Luteolin exhibited selective inhibition of MAO-A [MAO-A (IC_50_ = 8.57 μM), MAO-B (IC_50_ > 100 μM)] and is a selective antagonist of D_4_R (IC_50_ = 39.6 μM) and vasopressin receptor 1A (V_IA_R) (IC_50_ = 19.5 μM) calculated from a dose–response curve.

Isoliquiritigenin (**99**), isolated from *Glycyrrhizae rhizoma*, is a potent MAO inhibitor (MAO-A IC_50_ = 0.68 μM, MAO-B IC_50_ = 0.33 μM), measured by Promega’s MAO-Glo assay system.^225^ Isoliquiritigenin also displays modulatory functions on dopamine receptors (D_1_R IC_50_ = 68.9 μM, D_3_R EC_50_ = 62.3 μM) measured by HTRF-based cAMP assay and on V_IA_R (EC_50_ = 78.6 μM) measured by a fluorescent Ca^2+^ influx assay.

## 6 Conclusion and Perspectives

Natural products are an invaluable resource as lead compounds in drug discovery, inspiring a countless number of drugs in the treatment of debilitating diseases and illnesses. Despite the prevailing notion highlighting the potential for dopamine receptor-targeted ligands as therapeutic agents for the treatment of numerous diseases and illnesses, currently available ligands have limited success due to inadequacy in selectivity and other pharmacological properties. Subtype-selective dopaminergic drugs are lacking in the market.

Among the natural product scaffolds described, aporphines, THPBs, and ergolines have received the most scientific attention in relation to their dopamine receptor targeting. However, the cularines and miscellaneous natural products described have been relatively underexplored in this regard.

The development of functionally selective dopamine receptor tools is an area where natural product templates can have a significant impact. The identification of β-arrestin-biased D_2_R antagonists from the aporphine scaffold makes for a promising outlook in that direction.

Compared to the one-target, one-disease paradigm, polypharmacology enables compounds to have enhanced efficacy and safety through synergetic interactions. There is clear evidence that some diseases with complex etiology and/or symptomatology may be remedied optimally by drugs with multiple pharmacological actions. As privileged dopamine receptor-targeted scaffolds, aporphines, THPBs, and ergolines, in particular, offer a unique entry point to develop ligands with multireceptor activities.

While significant progress has been made in the development of dopamine receptor agents, it is clear that further progress is needed as the pharmacological properties can still be improved upon. Dopamine receptor-targeted natural products still have untapped potential in the development of therapeutic and investigative agents, and it is anticipated that they will continue to form a nucleus of inspiration for synthetic studies and drug discovery.

## Abbreviations Used

4-HPPA: 4-hydroxyphenylacetaldehyde
4HPAAS: 4-hydroxyphenylacetaldehyde synthase
AChE: acetylcholinesterase
ADHD: attention deficit hyperactivity disorder
ADME: absorption, distribution, metabolism, and excretion
BACE 1: beta-secretase 1
BBTHIQs: bis-benzyltetrahydroisoquinolines
BIAs: benzylisoquinoline alkaloids
BRET: bioluminiscence resonance energy transfer
BTHIQs: benzyltetrahydroisoquinolines
CBD: cannabidiol
CBT: cognitive behavioral treatment
CNS: central nervous system
d-GOV: d-govadine
D_1_R: dopamine receptor D_1_
D_2_R: dopamine receptor D_2_
D_3_R: dopamine receptor D_3_
D_4_R: dopamine receptor D_4_
D_5_R: dopamine receptor D_5_
DDCI: DOPA decarboxylase inhibitor
FDOPA: 6-[^18^F]fluoro-l-DOPA
GPCRs: G protein-coupled receptors
HTRF: homogeneous time-resolved fluorescence
HTS: high-throughput screening
l-GOV: l-govadine
l-ICP: isocorypalmine
l-SPD: (−)-stepholidine
l-THP: (−)-tetrahydropalmatine
LAT1: large neutral amino acid transporter
MAO: monoamine oxidase
NCS: norcoclaurine synthase
NDDs: neurodegenerative diseases
NMEs: new molecular entities
PAWS: protracted abstinence withdrawal syndrome
PNS: peripheral nervous system
SFSR: structure–functional–selectivity relationship
THC: Δ^9^-tetrahydrocannabinol
THPBs: tetrahydroprotoberberines
TYDC: l-tyrosine decarboxylase
V_IA_R: vasopressin receptor 1A
